# SNAI1-Mediated Epithelial-Mesenchymal Transition Confers Chemoresistance and Cellular Plasticity by Regulating Genes Involved in Cell Death and Stem Cell Maintenance

**DOI:** 10.1371/journal.pone.0066558

**Published:** 2013-06-17

**Authors:** Soyoung Lim, Astrid Becker, Andreas Zimmer, Jianrong Lu, Reinhard Buettner, Jutta Kirfel

**Affiliations:** 1 Institute of Pathology, University of Cologne, Cologne, Germany; 2 Institute of Molecular Psychiatry, University of Bonn, Bonn, Germany; 3 Department of Biochemistry and Molecular Biology, University of Florida College of Medicine, Gainesville, Florida, United States of America; 4 Institute of Pathology, University of Bonn, Bonn, Germany; Ghent University, Belgium

## Abstract

Tumor cells at the tumor margin lose epithelial properties and acquire features of mesenchymal cells, a process called epithelial-to-mesenchymal transition (EMT). Recently, features of EMT were shown to be linked to cells with tumor-founding capability, so-called cancer stem cells (CSCs). Inducers of the EMT include several transcription factors, such as Snail (SNAI1) and Slug (SNAI2), as well as the secreted transforming growth factor (TGFß). In the present study, we found that EMT induction in MCF10A cells by stably expressing SNAI1 contributed to drug resistance and acquisition of stem/progenitor-like character as shown by increased cell population for surface marker CD44^+^/CD24^−^ and mammosphere forming capacity. Using a microarray approach, we demonstrate that SNAI1 overexpression results in a dramatic change in signaling pathways involved in the regulation of cell death and stem cell maintenance. We showed that NF-κB/MAPK signaling pathways are highly activated in MCF10A-SNAI1 cells by IL1ß stimulation, leading to the robust induction in *IL6* and *IL8*. Furthermore, MCF10A-SNAI1 cells showed enhanced TCF/ß-catenin activity responding to the exogenous Wnt3a treatment. However, EMT-induced stem/progenitor cell activation process is tightly regulated in non-transformed MCF10A cells, as *WNT5A* and *TGFB2* are strongly upregulated in MCF10A-SNAI1 cells antagonizing canonical Wnt pathway. In summary, our data provide new molecular findings how EMT contributes to the enhanced chemoresistance and the acquisition of stem/progenitor-like character by regulating signaling pathways.

## Introduction

The ability of tumor cells to become invasive depends on the activation of an evolutionary conserved developmental process known as epithelial-to-mesenchymal transition (EMT) through which tumors cells lose homotypic adhesion, change morphology and acquire migratory capacity. Features of EMT have been observed in breast [1] and other tumor entities and inducers of EMT in cancer cell lines include transforming growth factor-ß1 (TGFß1), Wnt, Snail/Slug, Twist, Six1, Zeb1/2 [2]. The Snail family of transcription factors that includes Snail (SNAI1), Slug (SNAI2), and Smug is involved in physiological and cancer-associated EMT.

Recent reports indicate that EMT of tumor cells not only causes increased metastasis, but also contributes to the emergence of cancer stem cells (CSCs) in mammary epithelial cells. CSCs constitute a small minority of neoplastic cells within a tumor but they may generate tumors through the stem cell processes of self-renewal and differentiation into multiple cell types. Induction of an EMT in transformed human mammary epithelial cells were shown to yield cells with CSC-like character, such as CD44^+^/CD24^−^ phenotype and increased self-renewing capability [3]. Originally, CSCs are proposed to arise either from transformation of normal stem and progenitor cells, or through dedifferentiation of cancer cells. Importantly, transdifferential EMT process appears to promote dedifferentiation of cancer cells conferring many of the properties of the normal and neoplastic stem cell state.

In last decades, signaling pathways required for the maintenance of pluripotency in CSCs have been unraveled. Studies have demonstrated that active status of JAK/STAT, MAPK/ERK and PI3K/AKT was correlated with undifferentiated status of embryonic stem cells [4], [5]. Activation of NF-KB has been also detected in breast cancer stem-like cells [6]. Moreover, canonical Wnt/ß-catenin is involved in the stem cell renewal and implicated in a variety human cancer types including colon and breast carcinoma [7]. In addition to their role in the maintenance of pluripotency, these signaling pathways have been implicated in mediating drug resistance and survival of CSCs [8], [9].

Drug resistance of CSCs to chemotherapy is regarded as a major challenge to effective chemotherapeutic interventions against cancer [10]. While chemotherapy affects preferentially fast proliferating cancer cells, it might largely spare CSCs which divide slower and utilize very efficient drug resistance mechanisms. Due to their resistance to stress, cancer therapy leads to an increase in the number of CSCs which represents a potentially important mechanism of therapy failure and cancer recurrence including metastasis. Despite the importance of the drug resistance of CSCs, very little is known on how CSCs acquire very efficient drug resistance capacity.

In the present study, we examined whether EMT process in non-transformed MCF10A cells could enhance drug resistance and features of CSCs such as CD44^+^/CD24^−^ phenotype and mammosphere forming ability. To explore the underlying molecular mechanism how transdifferential EMT could contribute to the stem/progenitor process, Affymetrix microarray was performed using MCF10A cells stably expressing SNAI1. Here, we demonstrated that SNAI1-mediated EMT led to the dramatic change in signaling pathways involved in regulation of cell death and stem cell maintenance, thereby providing the cellular context favoring generation of CSCs.

## Materials and Methods

### Cell Culture and Reagents

MCF10A cells purchased from ATCC were a kind gift from Dr. M. Debald (Bonn medical school). MCF10A cells stably overexpressing SNAI1 were generated by Dr. J. Lu (University of Florida) [11]. The MCF10A-SNAI1 cells were obtained by transient transfection of MCF10A cells with pBeta-SNAI1-Flag, followed by puromycin selection. The pBeta plasmid modified from pCAGGS has a CMV enhancer, and the chicken beta-actin promoter followed by a chicken beta-actin intron sequence. This chimeric enhancer/promoter drives expression of SNAI1-Flag inserted into its downstream cloning site. The cells were cultivated in MEGM™ Mammary Epithelial Cell Growth Medium (Lonza, Germany) supplemented with 100 ng/ml cholera toxin (Sigma-Aldrich, Hamburg, Germany). MCF7 and MDA-MB-231 breast cancer cells were cultivated as described in Lim *et al*. [12]. Camptothecin (CPT), doxorubicin and trypan blue solution 0.4% were purchased from Sigma-Aldrich. Type I TGFß receptor kinase inhibitor (TGFRi) was obtained from Calbiochem (La Jolla, CA) and recombinant human IL1α/β from Humanzyme (Chicago, USA).

### FACS Analysis

Using Annexin V apoptosis detection kit (BD Biosciences, San Jose, CA), apoptotic cells were quantified, according to the manufactureŕs protocol. For determining cell surface marker CD44/CD24, harvested cells were incubated for 40 min on ice with α-CD44 (clone G44-26, BD Pharmingen, San Jose, CA) and α-CD24 (clone ML5, BD Pharmingen, San Jose, CA) antibodies diluted 1∶10 in 1% FBS/PBS. After washing, cells were analyzed using FACSCanto II flow cytometer (BD Biosciences, San Jose, CA) and FACSDiva software.

### MTT Assay

An 3-(3,4-dimethylthiazol-2-yl)-2,5-diphenyltetrazolium bromide (MTT) assay was performed, according to the manufactureŕs protocol (Roche, Mannheim, Germany).

### Immunofluorescence and Hoechst Staining

Immunofluorescence staining was performed as described in Park *et al*. [13]. The active caspase-3 was visualized using a rabbit polyclonal antibody against active caspase-3 (ab13847, Abcam, Cambridge, UK) and Alexa Fluor 488 Goat Anti-Rabbit IgG (Invitrogen, Karlsruhe, Germany). After immunofluorescence staining, cells were rinsed with PBS and stained with Hoechst 33342 (Invitrogen) for 10 min.

### Western Blot Analysis

Protein lysates were extracted from cells and blotted as described in Schulte *et al*. [14]. Following antibodies were used for WB: α-CDH1 and α-ß-catenin (1∶1000, 610181 and 610153, BD Bioscience), α-pAKT, α-WNT5A and α-PCNA (1∶1000, ab66138, ab72583 and ab29, Abcam, Cambridge, UK), α-β-ACTIN (1∶5000, Sigma-Aldrich), α-gamma-H2A.X, α-pp65, α-pp38, α-p38, α-pERK1/2, α-ERK1/2, α-AKT and α-pSTAT3 (1∶1000, 9718, 3033, 9211, 9212, 4377, 4372, 9272 and 9134, Cell Signaling Technology, Danvers, USA), α-SNAI1 and α-pSMAD2/3 (1∶200, sc-10432 and sc-11769, Santa Cruz Biotech, Santa Cruz, CA).

### Mammosphere Culture

Mammosphere forming assays were done according to the manufacturés protocol. Briefly, 15,000 cells were cultured with complete MammoCult medium (STEMCELL Technologies, Grenoble, France) in each well of a 6-well ultra-low adherent plate for 7 days and then mammopheres that were larger than 60 µm in size were counted. All experiments were repeated at least three times.

### Soft Agar Assay for Colony Formation

Soft agar assay was performed as described in Park *et al*. with some modifications [13]. Briefly, the base agar layer was prepared with 1% agar (Sigma-Aldrich) dissolved in 1.5 ml PBS on 6-well plates and then 1.5 ml of 0.3% agarose solution containing the cells (5,000 cells per well) were inoculated on top of the base agar layer. After allowing the solution to harden, 0.5 ml of the fresh medium was added to the top of the hard agar layer. After 3–4 weeks, agar plates were stained by Giemsa solution (Merck, Darmstadt, Germany) and colonies with more than 50 cells were counted.

### Microarray Anaylsis

Microarray experiments were performed with Affymetrix HuGene-1_0-st-v1 microarrays as described in Lim *et al.* using 1 µg total RNA. RNA integrity was assessed using the Agilent 2100 Bioanalyzer (Agilent Technologies, Böblingen, Germany). Analysis was performed according to the manufacturer’s recommendations. Hybridized arrays were washed and stained and finally scanned with the GeneChip scanner 3000 7G (Affymetrix, Santa Clara, USA). All array images were inspected for defects with the Expression Console software (Affymetrix, Santa Clara, USA). Normalization of the raw data by GC RMA algorithm, filtering and statistical data analysis was performed using GeneSpring GX 10.0 software (Agilent Technologies, Böblingen, Germany). To test for differential expression an unpaired *t*-test FDR corrected by Benjamini Hochberg was carried out between control and experimental samples. Genes were considered to have been changed by the treatment if statistical analysis reached p<0.01 and if the ratio of means exceeded a fold change of >2. To identify relevant and interrelated transcripts all detected candidate genes were hierarchically clustered and mapped.

The microarray data has been released into the GEO-database (accession number GSE 42388).

### Quantitative Real-time PCR (qRT-PCR)

Total RNA was isolated from cells using the RNeasy Mini kit (Qiagen, Hilden, Germany), and cDNA synthesis was performed using the SuperScript Reverse Transcription kit (Invitrogen). qRT-PCR was done as described by Lim *et al.* Expression values were normalized to the mean of 18S rRNA and PGK1 levels. Primers were designed using Universal ProbeLibrary Assay Design Center (Roche Applied Science, Minneapolis, USA). Primer sequences are available in the Supporting Information.

### TOPflash Luciferase Assay

To analyze the TCF/ß-catenin signaling activity in cells, 500 ng of TOPflash (Upstate, Danvers, USA), the reporter plasmid for TCF activity, and FOPflash (Upstate), the negative control, were transfected in cells using Lipofectamine (Invitrogen), respectively. The TOPflash plasmid contains TCF binding sites, while FOPflash contains mutant TCF binding sites. Two days after transfection, cells were treated with either 25 ng/ml Wnt3a ligand (R&D systems, Mineapolis, USA) or 1.25 nM TGFß RI kinase inhibitor (Merck, Darmstadt, Germany) for 6 h. TCF/ß-catenin activities were calculated by the ratio of TOP/FOP luciferase activities and relative light units were normalized to protein concentration.

### siRNA Transfection

Cells were transfected as described [12]. Control siRNA (Qiagen) or a pool of four *WNT5A* siRNAs (L-003939-00, Themo Scientific Dharmacon, Lafayette, CO, USA) were used in a range of 10–20 nM. To verify knock-down of WNT5A, RNA and protein were prepared after first round of siRNA transfection (3 days) and second round of siRNA transfection (6 days). To find out the effect of WNT5A knock-down on mammosphere forming capacity, cells were transfected with siRNAs and the transfection reagent (HiPerFect, Qiagen) for 3 days in adherent culture and then 15.000 cells were counted and subjected to the second siRNA transfection in mammosphere culture.

### Statistical Methods

Statistical significance was calculated using the Student’s *t*-test. Results were considered significant with a p-value <0.05 and marked with an asterisk.

## Results

### SNAI1-mediated EMT Induction Enhanced Resistance to Cell Death

**Figure 1:**
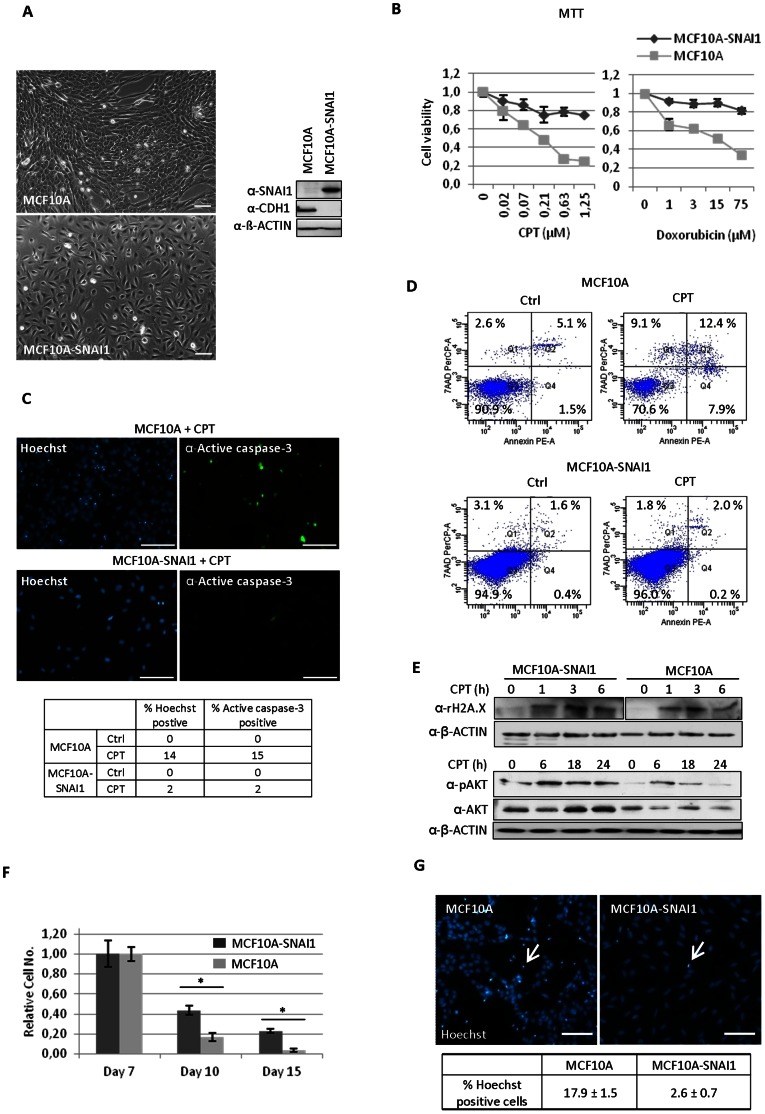
EMT generates cells with increased resistance to cell death. (A) MCF10A cells stably expressing SNAI1 (MCF10A-SNAI1) induced an EMT as shown by fibroblast-like appearance. Bar = 10 µm. Western blot analysis showed down-regulation of the epithelial marker, E-cadherin (CDH1) in SNAI1-overexpressing cells. (B) MCF10A-SNAI1 cells were more resistant to camptothecin (CPT) - and doxorubicin-induced cell death, as determined by MTT analysis. (C) MCF10A-SNAI1 cells were more resistant to the CPT-induced apoptosis as revealed by Hoechst staining and immunofluorescence using anti-active caspase-3 antibody. Positive cells for Hoechst or active caspase-3 staining were counted in the table. Bar = 100 µm. (D) Using Annexin V apoptosis detection kit, apoptotic cells were quantified upon CPT treatment (24 h). Viable cells are Annexin V-PE and 7-AAD-PerCP negative, and cells that are in early apoptosis are Annexin V-PE positive and 7-AAD-PerCP negative. Cells that are in late apoptosis or already dead are both Annexin V-PE and 7-AAD-PerCP positive. (E) To investigate the cellular response to CPT-induced DNA damage, western blot analysis was performed using antibody against γ-H2A.X, which is an indicator for DNA repair response. No difference in the accumulation of γ-H2A.X was observed in two cell lines for the indicated times, whereas MCF10A-SNAI1 cells showed higher AKT activation 6, 18 and 24 h after CPT treatment. (F) Serum-depletion for 10–15 days induced more cell death in MCF10A cells than MCF10A-SNAI1 cells as determined by a trypan blue assay. *P<0.05. (G) Hoechst positive cells upon serum-depletion were counted in three independent fields under a fluorescence microscopy. Bar = 100 µm.

Although the link between EMT in transformed cancer cells and the emergence of CSCs has been established, it is still unclear whether EMT itself has influence on the acquiescence of drug resistance of CSCs. For this purpose, we used MCF10A cells stably expressing SNAI1 (MCF10A-SNAI1) which underwent EMT [11]. MCF10A cells are immortalized, untransformed human mammary epithelial cells. While transient transfection of SNAI1 was not sufficient to induce EMT in MCF10A cells (Fig. S1), stable SNAI1 expression induced profound morphological changes, such as the dissolution of cell-cell junctions and fibroblast-like appearance (Fig. 1A). Western blot analysis showed that SNAI1 overexpression totally repressed the epithelial marker, E-cadherin (CDH1) (Fig. 1A).

To explore the possible association between SNAI1-induced EMT and chemoresistance, we incubated both cell lines with various concentrations of the chemotherapeutic drugs camptothecin (CPT) and doxorubicin for 24 h. MTT analysis showed that MCF10A-SNAI1 cells were more resistant to CPT- and doxorubicin-induced cell death than MCF10A cells (Fig. 1B) and this functional behavior was not caused by the empty control plasmid itself (pcDNA3.1(−)) (Fig. S2A). Immunofluorescence using anti-active caspase-3 and Hoechst staining revealed that MCF10A-SNAI1 cells had a higher apoptotic threshold to the CPT-induced apoptosis, while a significantly higher fraction of MCF10A cells were seen to be apoptotic (Fig. 1C). Likewise, Annexin V apoptosis detection revealed that almost all MCF10A-SNAI1 cells were viable (Annexin V PE-A and 7-AAD PerCP-A, negative) upon exposure to CPT for 24 h, whereas just 70.6% of MCF10A cells were viable and 7.9% of MCF10A cells in early apoptosis (Annexin V PE-A, positive and 7-AAD PerCP-A, negative) and 12.4% of MCF10A cells in late apoptosis or cell death (both Annexin V PE-A and 7-AAD PerCP-A positive) (Fig. 1D).

It has been reported that drug resistance of CSCs results from their efficient DNA repair system [9]. Since CPT induces DNA damage such as DNA double strand breaks by binding irreversibly to the DNA topoisomerase I complex, we investigated whether MCF10A-SNAI1 cells exhibit a rapid and efficient DNA repair capacity in a similar way like CSCs. Therefore, we analyzed the accumulation of phosphorylated forms of H2A.X, known as γ-H2A.X, an indicator for DNA damage response, at different time points. However, no significant difference was found in either kinetics or amounts of the phosphorylated forms of H2A.X (Fig. 1E). On the contrary, MCF10A-SNAI1 cells showed higher AKT activation upon CPT treatment, suggesting that SNAI1 overexpression increased pro-survival signaling under these stress conditions.

Since chemotherapeutic drugs affect preferentially fast proliferating cells and MCF10A-SNAI1 cells exhibit lower proliferation rate than MCF10A cells [15], we investigated whether MCF10A-SNAI1 cells also show resistance to cell death induced by the withdrawal of survival factors. Trypan blue assays showed that serum-depletion for 10–15 days induced more cell death in MCF10A than MCF10A-SNAI1 cells (Fig. 1F). In line, the number of Hoechst positive cells upon serum-depletion for 10 days was higher in MCF10A cells (17.9%) compared to MCF10A-SNAI1 cells (2.6%) (Fig. 1G).

### EMT Increased Epithelial Plasticity but no Tumorigenicity

**Figure 2:**
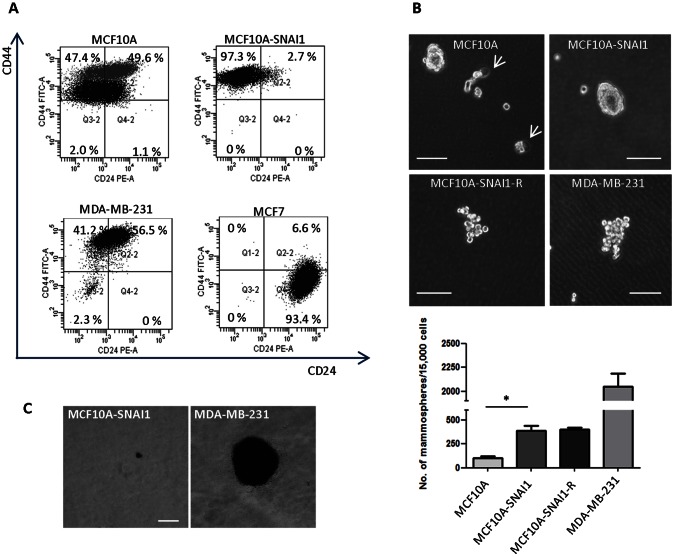
EMT generates CSC-like but not tumorigenic cells. (A) Cell population for surface marker CD44^+^/CD24^−^ which is associated with breast cancer stem/progenitor cell features was increased in MCF10A-SNAI1 cells. MDA-MB-231 cells and MCF7 cells were used as positive controls for CD44^+^ and CD24^−^, respectively. (B) MCF10A-SNAI1 cells showed increased mammosphere formation, while MCF10A cells underwent apoptotic death as shown by arrows. Subpopulation of MCF10A-SNAI1 cells (MCF10A-SNAI1-R) which survived the 5 nM doxorubicin-induced cellular toxicity showed no increased mammosphere forming ability. MDA-MB-231 cells were used as a positive control. Only mammospheres larger than 60 µm were counted. Bar = 100 µm. *P<0.05. (C) *In vitro* soft agar colony formation assay revealed that MCF10A-SNAI1 cells are non-transformed cells. MDA-MB-231 cells were used as a positive control. Bar = 100 µm.

In addition to the therapeutic resistance, CSCs have been reported to exhibit specific surface markers such as CD44^+^/CD24^−^ in breast cancer [16]. This phenotype alone may not be the most appropriate surface marker of CSCc, however, it is highly accepted that CD44^+^/CD24^−^ cells exhibit undifferentiated basal/mesenchymal cell properties, thereby being more enriched with the CSCs, while CD44^+^/CD24^+^ exhibit more differentiated basal/epithelial cell properties [17]. To determine whether the EMT undergone MCF10A-SNAI1 cells could increase the cell population that is enriched with CSCs, we conducted flow cytometry analysis to sort the cells based on the cell surface marker CD44 and CD24. Cell population for surface marker CD44^+^/CD24^−^ was increased in MCF10A-SNAI1 cells (97.3%), while MCF10A cells have 49.6% of CD44^+^/CD24^−^ subpopulation (Fig. 2A). Transfection of the plasmids itself led to only slight change in CD44^+^/CD24^−^ subpopulation (Fig. S2B). MDA-MB-231 cells and MCF7 cells were used as a positive control for CD44^+^ and CD24^+^, respectively.

The existence of progenitor cells can be assessed by a mammosphere formation assay. Primary human breast cell lines are capable of generating non-adherent spheres and normal or cancer stem/progenitor cells can be enriched in these non-adherent spheres [18]. Hence, we compared the mammosphere forming ability of the both cell lines. Significantly, MCF10A-SNAI1 cells formed 3-4 fold more mammospheres than MCF10A cells, while MCF10A cells underwent apoptotic death as shown by arrows (Fig. 2B). The subpopulation of MCF10A-SNAI1 cells (MCF10A-SNAI1-R) which was resistant to 5 nM doxorubicin exposure and survived the doxorubicin-induced cellular toxicity, was subjected to mammosphere assay. MCF10A-SNAI1-R cells formed looser and less rounded spheres similar to that of MDA-MB-231 cells but showed no increased mammosphere forming ability. This indicates that drug treatment could not enrich the cells with mammosphere forming ability and the emergence of drug-resistance subpopulation is the subsequent event upon SNAI1-induced EMT.

It has been reported that pluripotent cells have several cancer-resembling properties [19]. Therefore, we tested whether forced stable SNAI1 expression in MCF10A cells can induce tumorigenicity. *In vitro* soft agar colony formation assay revealed that MCF10A-SNAI1 cells could not form colonies greater than 100 µm, whereas MDA-MB-231 cells formed many colonies in soft agar suspension culture (Fig. 2C). In addition, in a xenograft nude mouse model, subcutaneous injection of MCF10A-SNAI1 cells did not form tumors, while tumors arose when an equal number of MDA-MB-231 cells were injected into mice (unpublished data). Taken together, induction of EMT in immortalized non-tumorigenic mammary epithelial cells creates populations of cells that are enriched for cancer stem-like cells as displayed by cell-surface marker expression and mammosphere formation. However, these cells were non-transformed and non-tumorigenic.

### SNAI1-mediated EMT Resulted in a Dramatic Change in Signaling Pathways

To explore the underlying molecular mechanism of drug resistance and enhanced plasticity by EMT we next analyzed transcriptome changes resulting from SNAI1 overexpression using a microarray approach. Comparing three independently reproduced transcriptomes of untransfected MCF10A cells and the SNAI1 overexpressing MCF10A-SNAI1 cells, respectively, we identified 966 genes with a fold change >2 (p-value <0.01). The microarray data were released into the GEO-database for public access (accession number GSE42388).

**Figure 3:**
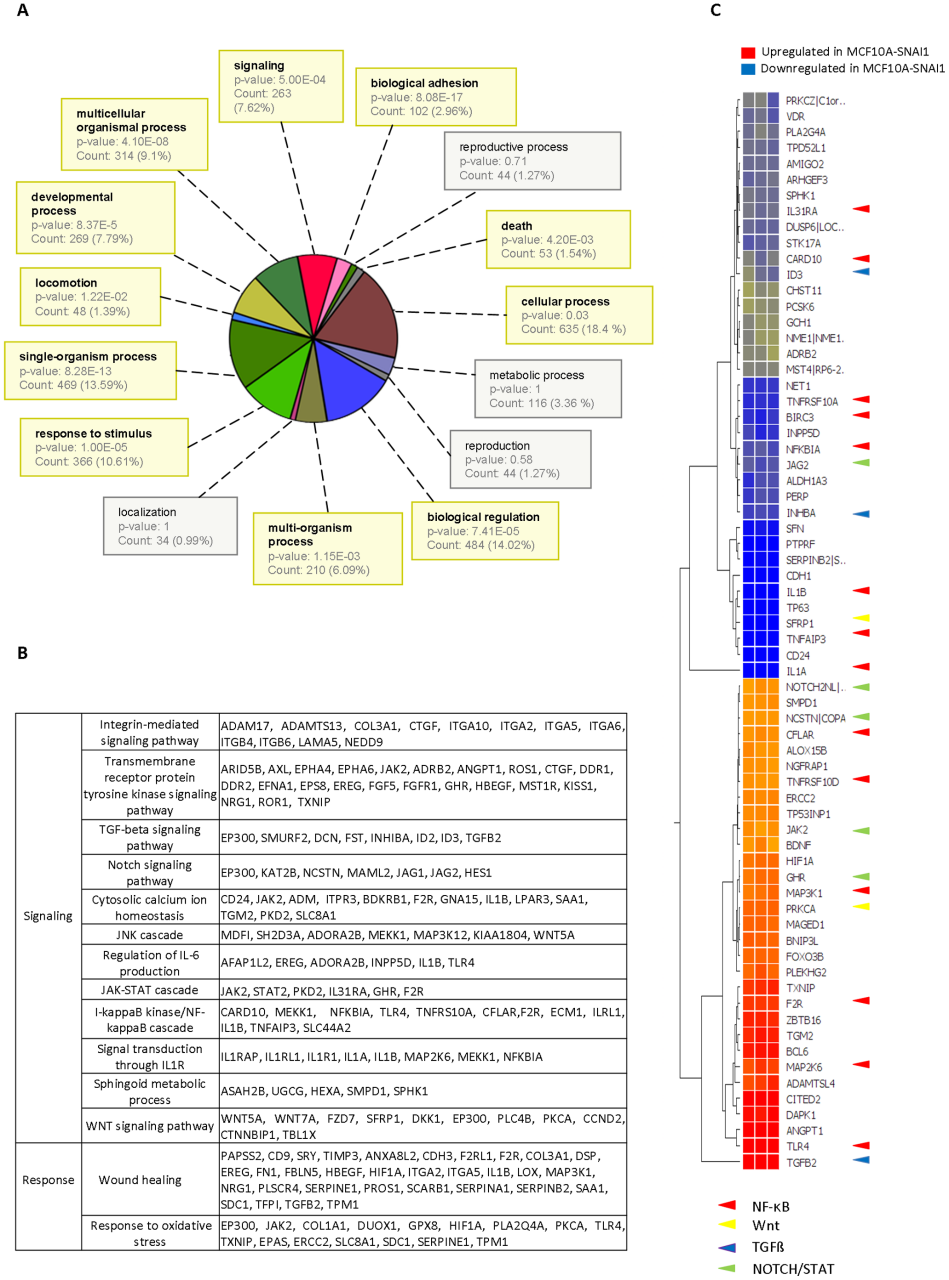
SNAI1 overexpression in MCF10A cells induced a dramatic change in gene expression profile. (A) The microarray discovered 966 genes differentially expressed in MCF10A and MCF10A-SNAI1 cells (fold change >2, *p*-value <0.01). 966 genes were subjected to GO analysis using Gene Spring GX program to gain insights on biological functions of the genes. Majority of differentially regulated genes were shown to be involved in signaling. FDR-corrected p-values were displayed and all significantly enriched processes were highlighted by bold characters. (B) Using the functional annotation clustering tool available in DAVID bioinformatics database, genes were classified into functionally related gene groups. Selected sets of genes under the categories like signaling and response were displayed. (C) In the heat map, genes involved in GO term, regulation of cell death were shown. Many of them were revealed to be associated with signaling pathways like NF-κB (red arrow), Wnt (yellow arrow), TGFß (blue arrow), and Notch/STAT (green arrow).

To gain insights into biological functions of changed genes, 966 genes were subjected to GO analysis using Gene Spring GX program. As expected, genes involved in `biological adhesion` including integrin complex and cell-cell junction, were highly enriched as shown in the diagram (Fig. 3A). Interestingly, genes related to `signaling` and `death` were greatly affected by SNAI1 overexpression. Subsequent analysis with the functional annotation clustering tool, available in DAVID bioinformatics database, revealed that integrin-mediated signaling pathway, transmembrane receptor protein tyrosine kinase and MAPKKK cascade had high enrichment scores in that order. A list of pathways showing significantly regulated genes was depicted in Fig. 3B including TGFß, Notch, NF-κB, IL1R and Wnt signaling. Highly enriched pathways further included response to stress (more detailed, wound healing and response to oxidative stress), suggesting that MCF10A-SNAI1 cells might have an advantage over stress-induced cell death through enhanced defence mechanism (Fig. 3B). Importantly, many genes that were associated with signaling pathways were also revealed to be related to the regulation of cell death like NF-κB (red arrow), Wnt (yellow arrow), TGFß (blue arrow), and Notch/STAT (green arrow) (Fig. 3C). This finding strengthened the idea of a potential role of these signaling pathways in the regulation of cell death.

### IL1ß Treatment Lead to the Production of IL6 and IL8 by Activating MAPK/NF-κB Signaling Pathways in MCF10A-SNAI1 Cells

**Figure 4:**
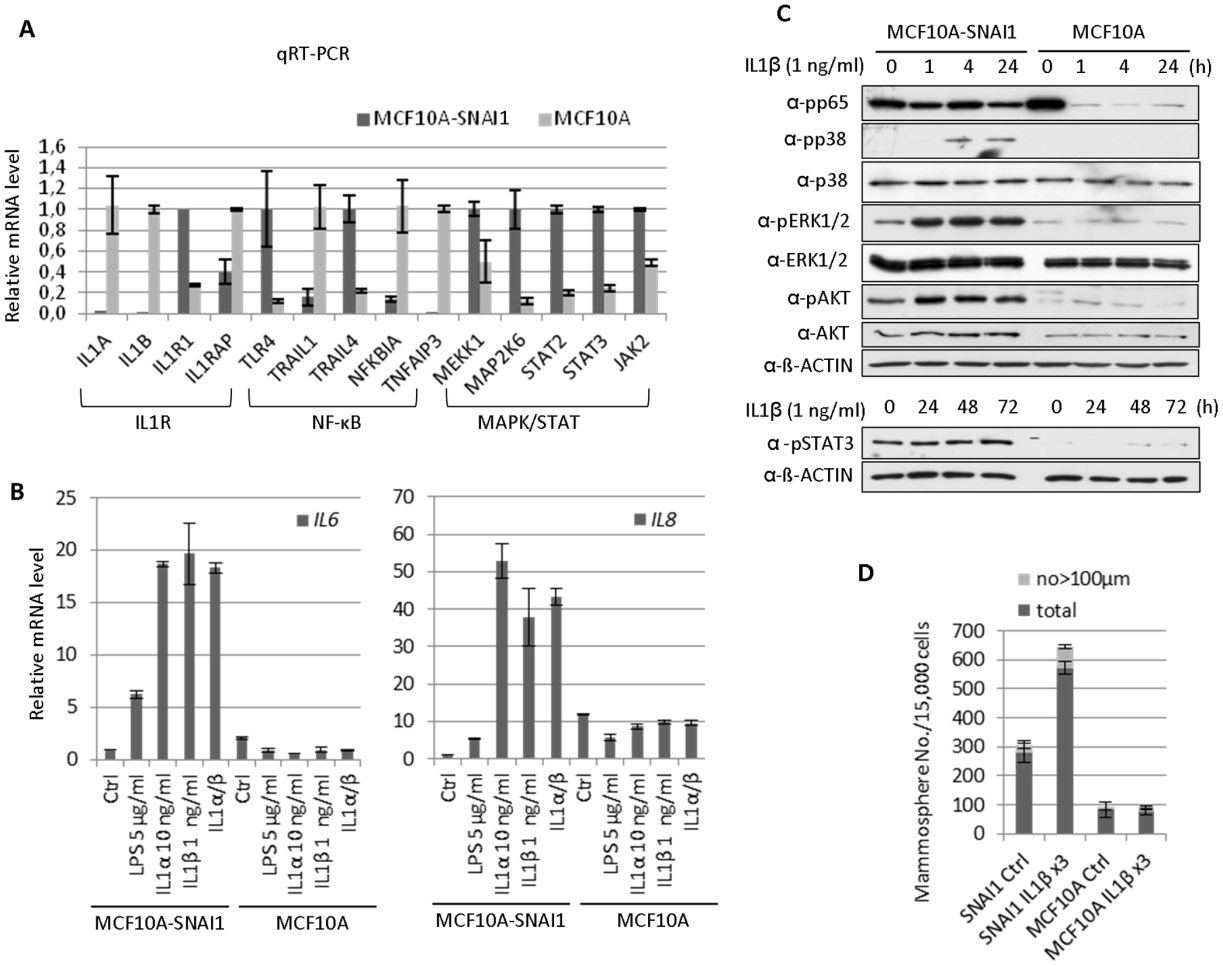
Differential gene expression pattern in NF-κB signaling and significant responses to IL1ß in MCF10A-SNAI1 cells. (A) Quantitative real-time PCR analysis confirmed the differential expression of genes revealed by microarray. (B) Treatment of LPS, IL1α, ILβ or combination of IL1α and ILβ increased *IL6* and *IL8* mRNA level in MCF10A-SNAI1 cells but not in MCF10A cells as analyzed by qRT-PCR. (C) WB analysis demonstrated that IL1β incubation leads to the activation of NF-κB, p38, ERK1/2 and AKT in MCF10A-SNAI1 cells but not in MCF10A cells. Constantly activated STAT3 was detected in MCF10A-SNAI1 cells regardless of IL1β addition. (D) Prolonged incubation of MCF10A-SNAI1 cells with IL1ß augmented the mammosphere formation ability.

Since microarray analysis revealed that NF-κB, IL1R and MAPK/STAT pathways were strongly affected by SNAI1 overexpression (Fig. 3B), we examined the role of these signaling pathways in acquired self-renewal capacity in MCF10A cells. Firstly, we validated the differential expression of genes related to NF-κB, IL1R and MAPK/STAT pathway by qRT-PCR. Steady-state transcript levels of IL1 receptor proteins (*IL1R1* and *IL1RL1*) or LPS receptor (*TLR4*), or signal transducers like *MEKK1, MAP2K6, STAT2, STAT3* and *JAK2* were highly up-regulated in MCF10A-SNAI1 cells, whereas IL1 ligands (*IL1A* and *IL1B*) or NF-kB inhibitors (*NFKBIA* and *TNFAIP3*) were greatly down-regulated (Fig. 4A). Since up-regulation of receptors/signal transducers may induce signal amplification, we analyzed the biological consequences of differential gene expression in SNAI1 over-expressing cells. In agreement with the up-regulation of IL receptor proteins and LPS receptors, treatment of LPS, IL1α, ILβ or combination of IL1α and ILβ increased dramatically *IL6* and *IL8* mRNA level in MCF10A-SNAI1 cells but not in MCF10A cells (Fig. 4B). Other NF-κB target genes such as *COX2* or *NOS2* were not changed upon ILs stimulation (unpublished data).

In addition, WB analysis demonstrated that IL1β incubation lead to the activation of NF-κB, p38, ERK1/2 and AKT in MCF10A-SNAI1 cells but not in MCF10A cells (Fig. 4C). Considering that MEKK1 phosphorylates ERK1 and MAP2K6 phosphorylates p38 MAP kinase, it seemed that up-regulation of *MEKK1* and *MAP2K6* in MCF10A-SNAI1 renders a greater sensitivity to the inflammatory stimuli, leading to the prolonged activation of NF-κB. Secondary, down-regulation of NF-κB inhibitors like *NFKBIA* and *TNFAIP3* might contribute to the persistant NF-κB activation in MCF10A-SNAI1 cells.

PI3K activity has a prominent role in mediating drug resistance and CSC expansion and maintenance [20]. Here, we could detect intense basal activation of AKT as well as increased AKT phosphorylation upon IL1ß treatment in MCF10A-SNAI1 cells (Fig. 4C).

Due to their contribution to maintenance and a self-renewal capacity of breast CSCs, induction of *IL6* and *IL8* by IL1ß in MCF10A-SNAI1 is of note. Since JAK/STAT genes were up-regulated in MCF10A-SNAI1 and IL6 activates STAT3, we investigated, whether a long-term incubation of IL1ß might increase STAT3 activation through up-regulation of *IL6*. However, WB analysis showed that STAT3 was constantly activated in MCF10A-SNAI1 cells regardless of IL1β addition (Fig. 4C). It appears that STAT3 activation is accomplished by receptor tyrosine kinase pathway in MCF10A-SNAI1 cells. Prolonged incubation of MCF10A-SNAI1 cells with IL1ß augmented the mammosphere formation ability, while MCF10A cells were not affected to IL1ß treatment, indicating the potential contribution of NF-κB signaling pathway to self-renewal capacity (Fig. 4D).

### Wnt5a and TGFß Signaling Antagonized Canonical WNT Pathway Blocking Mammosphere Formation in MCF10A-SNAI1 Cells

**Figure 5:**
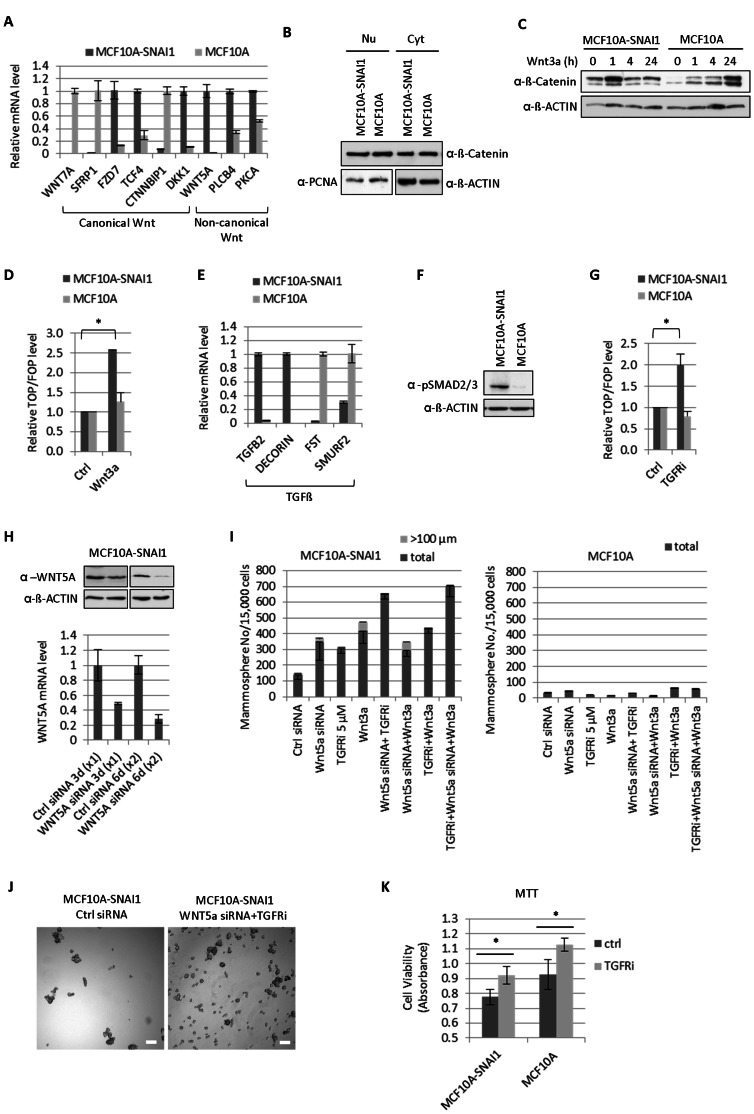
Wnt5a and TGFß signaling antagonized canonical WNT pathway blocking mammosphere formation in MCF10A-SNAI1 cells. (A) The expression of genes involved in canonical and non-canonical Wnt signaling pathway was analyzed by qRT-PCR. (B) No significant difference in nuclear/cytosol distribution of ß-catenin was observed upon SNAI1 expression. PCNA was used as a loading control for nuclear extract. (C) Wnt3a (25 ng/ml) resulted in the more rapid stabilization and activation of ß-catenin in MCF10A-SNAI1 cells. (D) Treatment of Wnt3a ligand (25 ng/ml) for 6 h increased TCF/ß-catenin activity in MCF10A-SNAI1 but not in MCF10A cells as shown by relative TOP/FOP ratios. *P<0.05. (E) The distinct expression of genes in TGFβ signaling pathway was analyzed by qRT-PCR. (F) Basal activation of SMAD2/3 indicates enhanced TGFβ level in MCF10A-SNAI1. (G) Treatment of TGFRi (1.25 nM) for 6 h increased TCF/ß-catenin activity in MCF10A-SNAI1 but not in MCF10A cells as shown by relative TOP/FOP ratios. *P<0.05. (H) Knock-down of *WNT5A* was confirmed by WB and qRT-PCR after first round of siRNA transfection (3 days, x1) and second round of siRNA transfection (6 days, x2), respectively. (I) Knock-down of *WNT5A,* treatment of TGFRi/Wnt3a or combinations of those led to the increased mammosphere formation in MCF10A-SNAI1 cells but not in MCF10A cells. (J) Microscopic image of mammospheres. Bar = 100 µm. (K) The exposure of cells to TGFRi (1.25 nM) for 10 days under the serum-depletion condition increased the resistance to cell death induced by the withdrawal of survival factors as shown by MTT assay. *P<0.05.

Our microarray analysis exhibited that genes related to the canonical Wnt and non-canonical Wnt/Ca^2+^ signaling were highly affected upon SNAI1 overexpression. While canonical Wnt signaling plays a crucial role in the maintenance of stem cell program, non-canonical Wnt/Ca^2+^ pathway antagonizes the canonical WNT signaling pathway by activating PLCB4/CamKII/NLK cascade [21]. qRT-PCR analysis showed that components of non-canonical Wnt signaling pathway such as *WNT5A*, *PLCB4* and *PKCA*, were up-regulated, while the expression pattern of canonical Wnt signaling players was more complex. The canonical Wnt ligand, *WNT7A* was strongly repressed, while *FZD7* and *TCF4* were strongly up-regulated in MCF10A-SNAI1 cells. In addition, Wnt antagonists like *SFRP1* and *DKK1* were down- and up-regulated, respectively (Fig. 5A).

To investigate whether the differential expression of Wnt signaling components has an influence on the activation status of canonical Wnt pathway in MCF10A-SNAI1 cells, we analyzed the nuclear translocation of ß-catenin by detecting ß-catenin protein in nuclear extracts. However, no increase in nuclear ß-catenin level was observed upon SNAI1 overexpression (Fig. 5B). Next, we treated cells with a recombinant canonical Wnt ligand, Wnt3a. In MCF10A-SNAI1 cells, Wnt3a resulted in the rapid stabilization of ß-catenin after 1 h incubation, whereas MCF10A cells responded late to the Wnt3a (Fig. 5C). Using the TOPflash system, we performed the TCF/ß-catenin reporter assay. Similarly, treatment of Wnt3a ligand for 6 h increased TCF/ß-catenin activity in MCF10A-SNAI1 but not in MCF10A cells as shown by relative TOP/FOP ratios (Fig. 5D). These findings indicate that differential gene expression of canonical Wnt pathway might be responsible for more rapid and sensitive response to the Wnt3a stimulation in MCF10A-SNAI1 cells.

TGFß signaling pathway was also found to be differently regulated in MCF10A cells expressing SNAI1. TGFß signals through type I and type II TGFß receptors activate a number of downstream effector proteins like SMAD2/3 or TGFß activated kinase 1 (TAK1). qRT-PCR analysis displayed that *TGFB2* and TGFß interacting partner Decorin (*DCN*) were strongly up-regulated, while SMAD specific E3 ubiquitin protein ligase 2 (*SMURF2*) which interacts with SMAD2 and suppresses TGFß function was down-regulated in MCF10A-SNAI1 cells indicating enhanced TGFß signaling pathway in the cells (Fig. 5E). Indeed, basal activation of SMAD2/3 was observed in MCF10A-SNAI1 cells (Fig. 5F).

Since TGFß was found to repress canonical Wnt pathway by activating TAK1 and Nemo-like kinase (NLK) [21], we treated cells with the TGFR inhibitor (TGFRi) to test whether the blockage of TGFß signaling could activate canonical Wnt pathway. We could verify the activation of TCF/ß-catenin activity upon TGFRi exposure for 6 h in MCF10A-SNAI1 but not in MCF10A cells (Fig. 5G) indicating that the activated TGFß signaling in MCF10A-SNAI1 cells antagonizes canonical Wnt signaling.

To investigate the physiological consequences of *WNT5A* and *TGFB2* up-regulation in MCF10A-SNAI1, we treated cells with *WNT5A* siRNA and/or TGFRi. We could see siRNA-mediated knock down of *WNT5A* (Fig. 5H). Treatment of *WNT5A* siRNA, TGFRi or Wnt3a ligand enhanced the mammosphere formation capacity, respectively, in MCF10A-SNAI1 but not in MCF10A cells. This indicates that *WNT5A* and TGFß2 negatively regulate self-renewal program in MCF10A-SNAI1 cells (Fig. 5I). Combinations of Wnt3a, *WNT5A* siRNA and TGFRi enhanced greatly the capacity of mammosphere formation and the size of mammospheres (Fig. 5J). In addition, prolonged exposure of cells to TGFRi led to the increased resistance to cell death induced by the serum withdrawal as shown by MTT assay (Fig. 5K).

## Discussion

### EMT Empowers Cells with Enhanced Survival Capacity

Our finding demonstrated that SNAI1-induced EMT enhanced cellular resistance to stress-induced cell death and increased cellular plasticity in non-transformed MCF10A cells. We showed that EMT was accompanied by not only a change in structural proteins but even more a broad range of change in intracellular signaling networks involved in cell death and stem cell maintenance as summarized in a schematic overview (Fig. S3). GO analysis showed that genes related to the regulation of cell death were also highly enriched and many of them were associated with NF-κB/IL1R and JAK/STAT signaling pathways, suggesting a pivotal role of these signaling pathways in resistance to cell death. Activation of MAPKs such as ERKs and AKT in MCF10A-SNAI1 cells is also regarded to contribute to the cell survival in response to a wide variety of stimuli, including retrieval of growth factors and DNA damages [9], [22], [23].

Kurrey *et al.* have reported that SNAI1/2 directly regulates the repression of pro-apoptotic genes such as *PUMA, ATM* and *PTEN* under conditions of stress conferring resistance to the p53-mediated apoptosis [24]. However, without stress conditions expression changes of *PUMA, ATM* and *PTEN* were not detectable. Considering the broad change in signaling pathways in MCF10A-SNAI1, it is a tempting idea that MCF10A-SNAI1 cells might respond differently in response to apoptotic stimuli and repress the transcription of apoptotic genes to block thereby apoptosis. Taken together, the transcriptome changed by EMT program appears to empower epithelial cells with the biological traits needed not only for cell movement but also for the survival while transiting through the invasion-metastasis cascade and adaptation to the foreign microenvironment [25].

### EMT Contributes to Cellular Plasticity by Regulating Inflammatory Mediators

Although the connection between the EMT and the acquiescence of stem cell-like traits was established, it is still not clear how EMT provides the cellular context in favor to emergence of stem/progenitor cell on the molecular basis. Our study provides insights that cellular plasticity upon EMT is mediated through regulation of signaling pathways involved in stem cell maintenance like Notch, Wnt, PI3K/AKT, NF-κB/MAPK, and JAK/STAT3 [18], [26]–[29]. Especially, we have focused on the regulation of inflammatory mediators by EMT. We found that IL1R downstream signaling molecules including NF-κB/MAPK signal transducers were highly up-regulated thereby enabling MCF10A-SNAI1 cells to respond dramatically to exogenous IL1ß, producing high amounts of *IL6* and *IL8*. Recently, the pivotal role of IL1ß, IL6, and IL8 in CSC formation has been identified, supporting the links between inflammatory states and cancer development [30]. IL1, produced in response to infection or tissue injury results in activation of NF-κB and downstream targets IL6 and IL8. IL6, a direct regulator of breast CSC self-renewal activates STAT3 and NF-κB which secretes additional IL6 and IL8, generating a positive feedback loop between inflammatory cell and tumor cells [31]. Furthermore, IL6 activates Jagged1/Notch-1 and -3, well-known stem cell regulators [32]-[34]. In addition, IL8 functions as a stem cell regulator as shown by up-regulation of IL8 receptor, CXCR1, in breast CSCs and increased breast CSC self-renewal and tumor growth by exogenous IL8 [35]. Moreover, elevated levels of IL1, IL6 and IL8 were shown to be correlated with aggressive cancer behavior and poor prognosis [31], [36], [37].

### EMT-induced Stem/Progenitor Cell Activation Process is Tightly Regulated to Keep Cellular Homeostasis

It was noteworthy that in MCF10A-SNAI1 cells, *IL1A/B* was strongly down-regulated blocking signal transduction through IL1R in a normal state. Likewise, a strong down-regulation of *WNT7A* was observed in the cells. In line, activation of canonical Wnt signaling was constantly antagonized by the enhanced status of non-canonical WNT5A and TGFß pathways in MCF10A-SNAI1 cells, although the cells were able to react more rapidly and sensitive to the exogenous Wnt3a ligand, possibly due to up-regulation of receptor (*FZD7*)/transcription factor (*TCF4*) and down-regulation of antagonists (*SFRP1*, *CTNNBIP1*). More importantly, the exposure of cells to IL1ß/Wnt3a or the blockage of *WNT5A*/TGFß signaling could not dramatically increase the self-renewal capacity or tumorigenicity of MCF10A-SNAI1 cells, as we could not observe any colony formation on the soft agar from MCF10A-SNAI1 cells treated long-term with IL1ß or TGFRi (unpublished data). This indicates that a complex crosstalk of signaling pathways maintains homeostasis by using positive or negative feedback mechanism to guard against uncontrolled plasticity and neoplastic transformation upon EMT. Apparently, EMT is not sufficient to provide self-renewal ability and may require the collaborative actions of other signaling agent to acquire full traits of CSCs. Indeed, merely when already transformed cells with e.g. aberrant EGFR, HER/neu, or RAS mutations underwent EMT process, EMT could promote greatly tumorigenicity and generation of cancer stem cells [3]. Considering the role of EMT in a normal biological process like wound repair or tissue development, it is not surprising that transdifferential EMT endows cells with plasticity while governing strictly the balance between self-renewal capacity and differentiation.

### The Crosstalk between TGFß and WNT5A Signaling Antagonizes Self-renewal Program in MCF10A-SNAI1 Cells

Here, we found that up-regulated *WNT5A* and *TGFB2* act as negative players of stem cell maintenance program by antagonizing canonical Wnt pathway in MCF10A-SNAI1 cells. Blocking WNT5A/TGFß signaling enhanced mammosphere formation and TGFRi increased resistance to serum-depletion induced cell death. TGFß1/2 has been reported to have biphasic effect in carcinogenesis. While, in late stage, TGFß functions as oncogene by inducing EMT which contributes to the acquisition of the CSC phenotype and metastasis [38], in early stage, TGFß can suppress tumorigenesis by depleting the putative cancer stem/early progenitor cell population and promoting differentiation of the more commuted progeny [39]. Anti-proliferative effect of TGFß is mediated through the SMAD2/3-dependant pathway resulting in inhibition of cell cycle and promotion of apoptosis. In SMAD-independent manner, tumor suppressive functions of TGFß involve TAK1/NLK activation-mediated antagonism of Wnt/ß-catenin signaling limiting the stem cell population [40]. The crosstalk between TGFß and Wnt signaling was also shown in which, TGFß regulates directly the transcription of *WNT5A* which is able to activate TAK1/NLK through intracellular calcium concentration increase and calmodulin-dependent kinase (CamKII) activation [40], [41]. Detailed investigation of the interplay between these signaling cascades is an important task to be done in further study to verify antagonistic role of TGFβ/WNT5A in the self-renewal program in MCF10A-SNAI1 cells. Our gene expression profiling showed that genes related to cytosolic calcium ion homeostasis were also highly enriched upon SNAI1-induced EMT, giving evidence that up-regulated *WNT5A* might activate Ca^2+/^calmodulin-dependent TAK1/NLK axis antagonizing canonical Wnt signaling.

In summary, we demonstrated that EMT in non-transformed mammary epithelial cells does not confer tumorigenicity on itself, but plays an enhancer role in stem/progenitor processes by increasing resistance to death and cellular plasticity. SNAI1-mediated EMT resulted in a broad range of change in signaling pathways involved in the regulation of cell death and stem cell maintenance. Our data suggest that EMT-mediated transcriptome changes in NF-kB/MAPK, WNT and TGFß signaling network could provide the cellular context favoring generation of CSCs. We also report that EMT programs govern tightly the balance between self-renewal capacity and differentiation by antagonizing canonical Wnt signaling. Our findings suggest that targeting EMT process might contribute to find a way to overcome chemoresistance caused by the emergence of CSCs.

## Supporting Information

Figure S1 **Transient SNAI1 overexpression in MCF10A was not sufficient to induce EMT.** (A) No EMT-feature such as a spindle-like morphology was observed after transient transfection of MCF10A cells with pcDNA 3.1 (−)-SNAI1-HA (MCF10A-SNAI1-HA). For its transfected counterpart, cells were transfected with pcDNA 3.1 (−) plasmid (empty vector). Bar = 10 µm. (B, C) Transient transfection of MCF10A cells with pcDNA 3.1 (−)-SNAI1-HA resulted in successful overexpression of HA-tagged SNAI1 as shown by qRT-PCR and WB. However, MCF10A-SNAI1-HA cells did not exhibit the EMT-feature like loss of E-cadherin.
(TIF)

Figure S2 **Transient SNAI1 overexpression in MCF10A did not induce EMT-mediated cellular changes.** (A) No difference in chemoresistance behavior was found between untransfected, and empty vector- and SNAI1-HA expressing vector-transfected MCF10A cells. The untransfected cells were used as a reference for its transfected counterpart. (B) No significant change in cell population for surface marker CD44^+^/CD24^−^ was observed upon the transient transfection of MCF10A cells with empty and SNAI1-HA expressing vectors, respectively.
(TIF)

Figure S3 **Schematic overview of cell signaling regulation by SNAI1-mediated EMT in MCF10A cells.** SNAI1 overexpression increased cellular responses to exogenous IL1ß and Wnt3a by regulating transcriptional profiling of IL1R/NF-κB, Wnt and TGFß signaling cascades, leading to enhanced self-renewal capacity. SNAI1-induced EMT also generates a negative feedback loop that antagonizes stem cell program by regulating IL1α/β, Wnt7a and TGFß2.
(TIF)
