# Supplementary figures and images for: SNAI1-Mediated Epithelial-Mesenchymal Transition Confers Chemoresistance and Cellular Plasticity by Regulating Genes Involved in Cell Death and Stem Cell Maintenance
Source: PLoS One. 2013 Jun 17;8(6):e66558. doi: 10.1371/journal.pone.0066558 (PMC3684605; doi:10.1371/journal.pone.0066558)

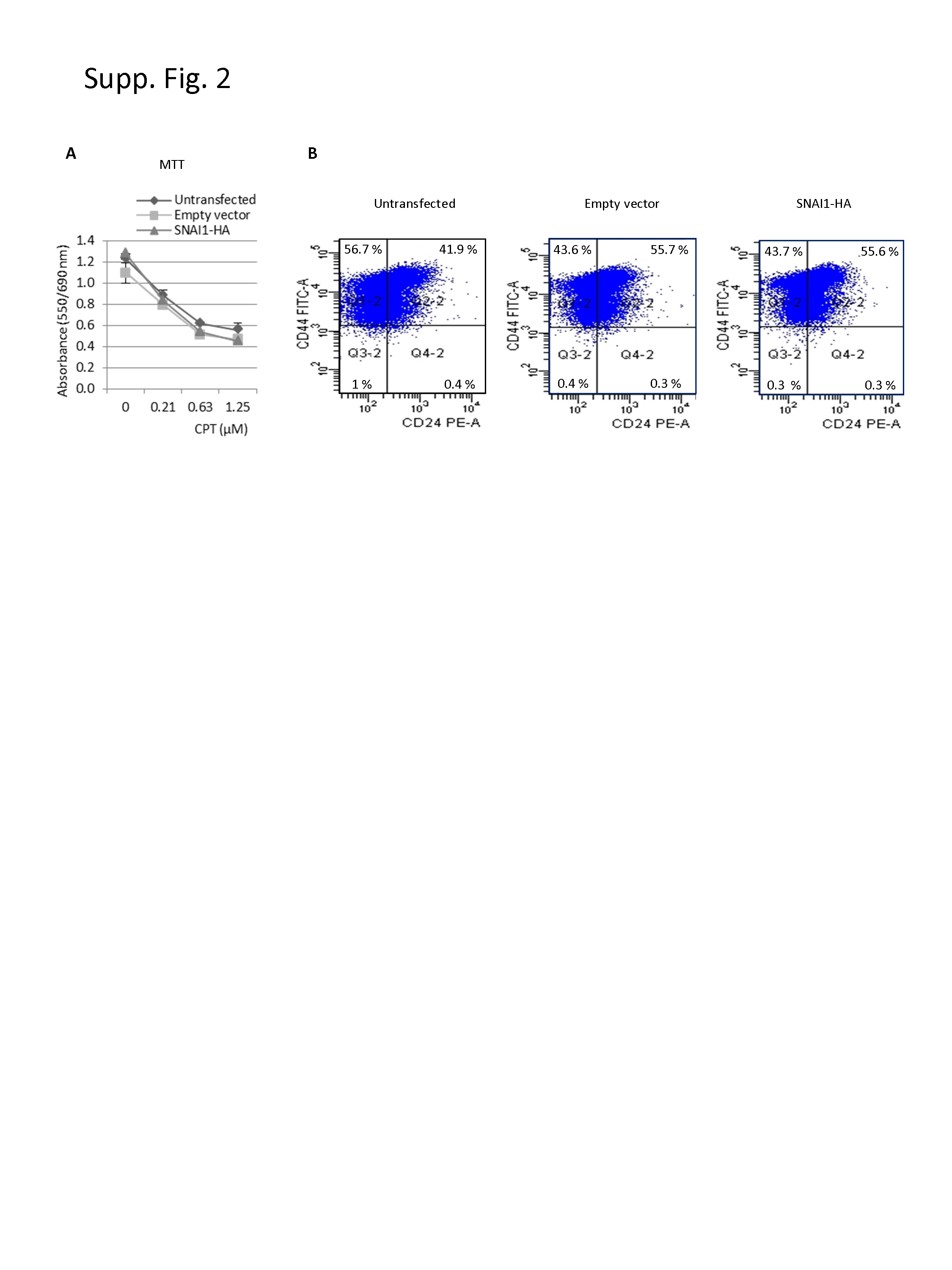

Supplement: Figure S2 — Transient SNAI1 overexpression in MCF10A did not induce EMT-mediated cellular changes. (A) No difference in chemoresistance behavior was found between untransfected, and empty vector- and SNAI1-HA expressing vector-transfected MCF10A cells. The untransfected cells were used as a reference for its transfected counterpart. (B) No significant change in cell population for surface marker CD44+/CD24− was observed upon the transient transfection of MCF10A cells with empty and SNAI1-HA expressing vectors, respectively. (TIF) [file pone.0066558.s002.tif]

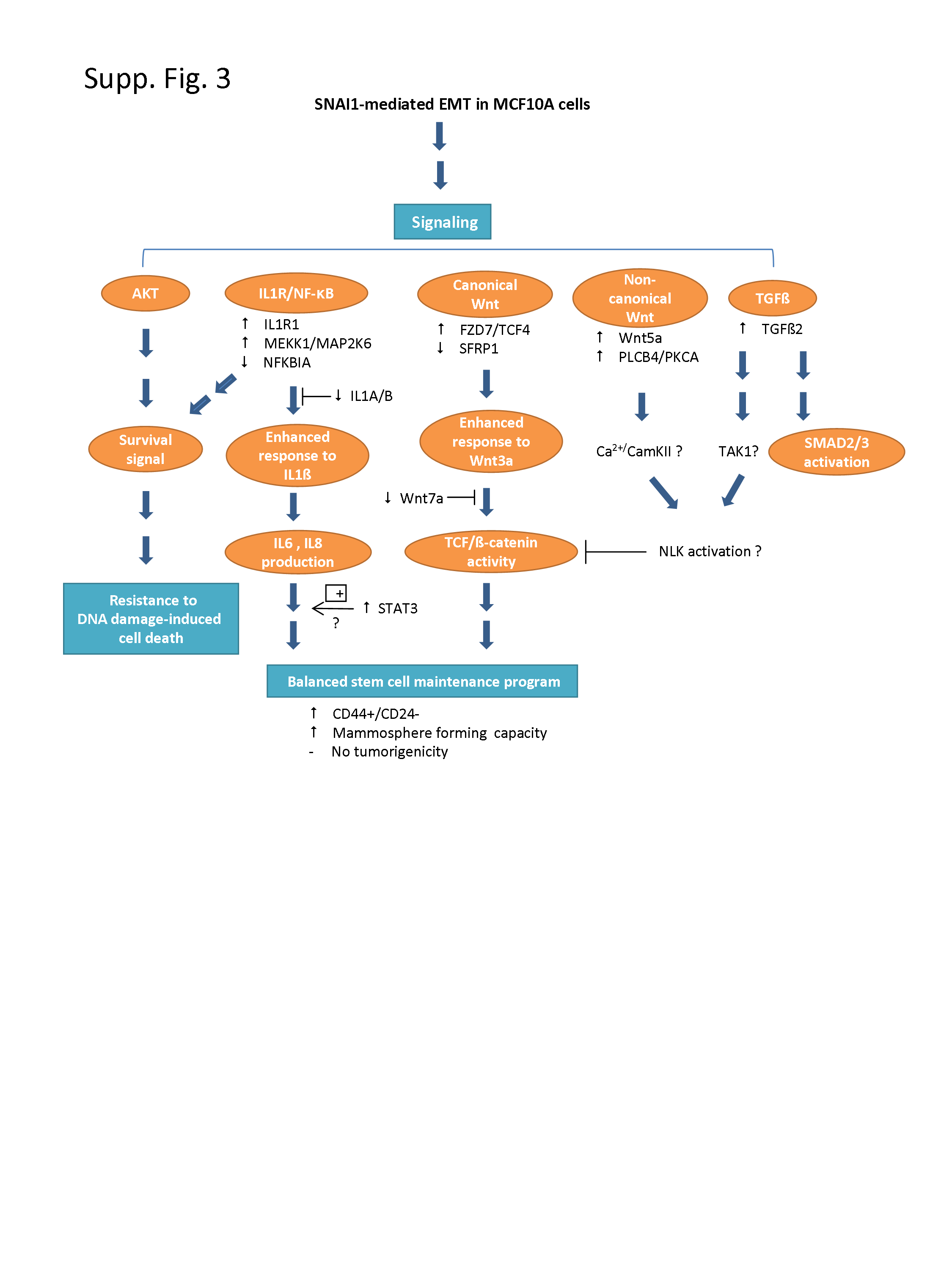

Supplement: Figure S3 — Schematic overview of cell signaling regulation by SNAI1-mediated EMT in MCF10A cells. SNAI1 overexpression increased cellular responses to exogenous IL1ß and Wnt3a by regulating transcriptional profiling of IL1R/NF-κB, Wnt and TGFß signaling cascades, leading to enhanced self-renewal capacity. SNAI1-induced EMT also generates a negative feedback loop that antagonizes stem cell program by regulating IL1α/β, Wnt7a and TGFß2. (TIF) [file pone.0066558.s003.tif]
